# Draft Genome Sequence of a Single Cell of SAR86 Clade Subgroup IIIa

**DOI:** 10.1128/genomeA.00030-12

**Published:** 2013-01-15

**Authors:** D. B. Rusch, M.-J. Lombardo, J. Yee-Greenbaum, M. Novotny, L. M. Brinkac, R. S. Lasken, C. L. Dupont

**Affiliations:** a Informatics Group, J. Craig Venter Institute, Rockville, Maryland, USA; b The Center for Genomics and Bioinformatics, Indiana University, Bloomington, Indiana, USA; c Department of Microbial and Environmental Genomics, J. Craig Venter Institute, San Diego, California, USA

## Abstract

SAR86 denotes a 16S clade of gammaproteobacteria that are ubiquitous in ocean surface waters. So far, SAR86 is resistant to cultivation; thus, little is known about the genome contents or physiology of this clade. Recently, four partial genome sequences for SAR86 subclades I and II were published. Here, we present the draft genome sequence of a single cell from SAR86 subgroup IIIa isolated from coastal waters in San Diego, CA.

## GENOME ANNOUNCEMENT

The 16S clade SAR86 has been observed in the microbial communities of each of the major oceans (1–5). Multiple subgroups are present within the SAR86 16S phylogeny, including the subgroups I, II, IIIa, and IIIb, though it is not known whether these are related to differences in ecophysiology (6). The sequencing of an ∼40-kbp bacterial artificial chromosome (BAC) and fosmid clones generated from a variety of locations revealed that some SAR86 genomes contain a gene that encodes a rhodopsin capable of generating a proton motive force (PMF) using light energy (proteorhodopsin [7–9]), with divergences between the subclades. Recently, two near-complete genome sequences from subclades I and II were assembled from metagenomic data and provided insight into the metabolic potential of SAR86 (10). The two subclades displayed distinct environmental distributions related to temperature, suggesting an ecophysiological link to a phylogenetic divergence, though representatives from other clades are necessary to make this conclusion.

Seawater was collected at the Scripps Institution of Oceanography (SIO) pier on 9 April 2009 at 8:30 a.m. The water temperature was 13.5°C, with a salinity of 33.5 practical salinity units (PSUs) and a chlorophyll fluorescence of 2.7 µg liter^−1^ (SIO Automated Shore station equipment, http://www.sccoos.org). Single cells were sorted and amplified using multiple displacement amplification (MDA) technology as described by Dupont et al. (10).

The MDA reaction mixture was sequenced on one lane of an Illumina GAIIx. The reads were trimmed with Trimmomatic, and iterative *de novo* assemblies were performed using the CLC Genomics workbench. The read set was normalized to reduce coverage biases caused by MDA. To aid with gap closure, high-identity (>95% nucleotide identity) paired-end metagenomic reads from the Global Ocean Sampling Project (11) were aligned to the contigs to help orient and order them. Gap closure was performed using PCR and Sanger sequencing. Annotation was performed using the Prokaryotic Annotation Pipeline (12) with visualization and manual curation in Manatee. We have assembled a genome of 7 scaffolds of 13 contigs totaling 1.40 Mbp, 1,442 putative coding sequences, 33 tRNAs, and 1 rRNA operon.

A phylogenetic analysis of the full-length 16S gene placed the single cell in SAR86 subclade IIIa, along with two BACs (accession no. AY033328 and AF268217); thus, we have named the genome SAR86_IIIb_sincell1. The genome contains many of the same features observed by Dupont et al. (10), including an overabundance of *tonB* receptors, a complete Emden-Meyerhof-Parnas glycolytic pathway, a green-light-absorbing proteorhodopsin, and the molecular machinery for dimethylsulfoniopropionate (DMSP) and glutathione assimilation. Like the other genomes, it also lacks several synthetic pathways, including those for vitamins and the carotenoids needed for light harvesting. Uniquely, SAR86_IIIb_sincell1 contains a hypervariable region with poor coverage in ocean metagenomes. This hypervariable region contains the genes involved in outer membrane modification, including those for putative glycosyltransferases, fucose acetylases, GDP-mannose dehydratases, and acetyltransferases. A similar scenario has been observed in the genomic islands of marine *Synechococcus* strains WH8102 and CC9311, where it has been suggested to alter the interactions with predators and phages (13, 14).

### Nucleotide sequence accession number.

This whole-genome shotgun project was deposited in NCBI under the accession no. AMWX00000000 (BioProject 170317).
